# *Ndufs4*^*−/−*^ mice: a testing ground for longevity interventions

**DOI:** 10.1007/s11357-025-01704-8

**Published:** 2025-06-05

**Authors:** Jackson Nuss, Matt Kaeberlein, Alessandro Bitto, Anthony S. Grillo

**Affiliations:** 1 Department of Laboratory Medicine & Pathology, School of Medicine, University of Washington, Seattle, WA USA; 2 Optispan, Tukwila, WA USA; 3 Department of Chemistry, University of Cincinnati, Cincinnati, OH USA

**Keywords:** Mitochondrial dysfunction, Interventions, Longevity, Vertebrate models

## Abstract

Mice missing the complex I subunit Ndufs4 of the electron transport chain are widely used as a leading animal model of Leigh syndrome, a pediatric neurodegenerative disorder that leads to premature death. More broadly, this animal model has enabled a better understanding of the pathophysiology of mitochondrial disease and mitochondrial dysfunction in sporadic disorders. Intriguingly, longevity interventions are very effective at treating symptoms of disease in this model. Herein, we introduce the model and its notable features that may help provide insights in longevity research. We performed a retrospective analysis of historical data from our laboratories over the past 10 years regarding the use of this animal model in aging studies, the manifestation and progression of mitochondrial disease, and factors that influence their premature death. We observed a correlation between weight and lifespan in female animals and a sex-independent correlation between the onset of clasping, a typical neurodegenerative symptom, and overall survival. We observed a sexual dimorphism in lifespan with female mice being more resilient despite a similar age of onset of disease symptoms. Lastly, we report increased lifespan and delayed onset of disease symptoms following treatment with 17-alpha-estradiol, a non-feminizing estrogen which can extend lifespan in genetically heterogeneous mice. This analysis serves as a useful guide for researchers utilizing this animal in the discovery of effective interventions for longevity and to prevent the onset of disease. It suggests there may be unprecedented underlying sex-specific differences in patients with Leigh syndrome and further strengthens the connection between normative aging and mitochondrial dysfunction.

**Supplementary Information:**

The online version contains supplementary material available at 10.1007/s11357-025-01704-8.

## Introduction

Many genetic diseases are caused by aberrant mitochondrial function [1]. Mitochondrial dysfunction is similarly thought to be pathogenic in many disorders of normative aging, such as Alzheimer’s disease (AD), Parkinson’s disease (PD), heart disease, sarcopenia, and diabetes [1]. The prevalence of mitochondrial dysfunction contributing to the progression of these diseases has led to its recognition as a central hallmark of aging [2]. Diverse genetic diseases of mitochondrial dysfunction such as Leigh syndrome (LS), mitochondrial encephalopathy with lactic acidosis and stroke-like episodes (MELAS), Friedreich’s ataxia (FA), and maple syrup urine disease (MSUD), exhibit similar neurodegenerative pathology and metabolic dysregulation as observed in many age-related disorders. More specifically, decreased respiratory capacity due to inefficient or defective utilization of oxygen is often associated with many of these genetic or age-progressive diseases [3].

LS is a neurometabolic mitochondrial disease affecting 1:50,000 births, with a significantly higher prevalence of ~ 1:5,000 in select populations [4]. Mice missing the complex I subunit NDUFS4 (*Ndufs4*^*−/−*^) are a leading mammalian model of LS. These mice exhibit similar pathology to the human disease, such as neurological lesions of the brain stem and basal ganglia, decreased motor coordination, seizures, and premature death [5]. Aging interventions that extend lifespan in healthy mice through the National Institute on Aging (NIA) Interventions Testing Program (ITP), such as the mechanistic target of rapamycin (mTOR) inhibitor rapamycin [6, 7] and the alpha-glucosidase inhibitor acarbose [8], extend lifespan in *Ndufs4*^*−/−*^ mice. Similarly, other longevity treatments such as alpha-ketoglutarate and NAD^+^ precursors [9] can improve survival or disease symptoms in this model. Lastly, reduced oxygen tension suppresses symptoms and greatly extends survival in *Ndufs4*^*−/−*^ mice [10, 11], similar to its effects on increased longevity in invertebrate models [12, 13] and a mouse model of accelerated aging [14]. This evidence suggests that the *Ndufs4*^*−/−*^ mouse model may have untapped potential to provide insights into the pathogenic effects of mitochondrial dysfunction in age-related disorders and aging itself. It may also streamline the discovery of novel interventions to treat a wide array of age-related human diseases associated with Complex I dysfunction.

Herein, we describe this mouse model from a geroscience perspective using historical data generated over the last ten years. We report statistics for weight, onset of disease symptoms, lifespan, and correlations among these parameters. We describe subtle, yet significant, sex-specific differences consistent with normative aging in mammals. We present data showing that thermoneutrality does not influence lifespan and disease progression in this model. Lastly, we present evidence that 17-alpha-estradiol increases lifespan and delays onset of disease in this model and has some sex-specific effects on disease onset. These data further support the utility of this model as a short-lived mammalian system for research and discovery in geroscience [8, 15].

## Methods

### Animal experiments

C57BL/6 N *Ndufs4*^*−/−*^ mice and *Ndufs4*^+*/*+^ littermates were generated by breeding heterozygous pairs in the University of Washington Foege/Animal Research and Care Facility (ARCF) animal vivarium. All animals were genotyped before post-natal (p.n.) day 21, and littermate pairs of different genotypes were randomly assigned to treatments near p.n. day 21. Mice that weighed less than 6.5 g at p.n. day 21 were kept in the breeder cage until they reached that weight or p.n. day 28, whichever occurred first. All animals were maintained in the Foege/ARCF animal vivarium at the University of Washington and housed in groups of two to five in either Allentown JAG 75 or Allentown NexGen Mouse 500 cages, in temperature-controlled rooms (25 °C) on racks providing filtered air and filtered, acidified water. Animals were housed on a 14-h light, 10-h dark cycle. Animals were fed Pico Lab Diet 20 5053. Diet was supplemented with 14.4 ppm 17-alpha-estradiol mixed in the same chow (Hello Bio, Princeton NJ) for treated animals. For survival curves and monitoring of neurological symptoms, animals were monitored two to three times a week until onset of symptoms and then monitored daily until endpoint. Animals were euthanized when they showed any of the following signs: (1) loss of over 30% of the maximum weight recorded; (2) inability to eat or drink; (3) severe lethargy, as indicated by a lack of response such as a reluctance to move when gently prodded; and (4) severe respiratory difficulty while at rest, indicated by a regular pattern of deep abdominal excursions or gasping.

### Statistical analysis

All data were analyzed using the GraphPad Prism software. *p* values of 0.05 or lower were considered statistically significant. In general, data are presented as the mean ± SEM. See figure legends for the specific tests used.

### Ethics statement

All animal experiments were reviewed and approved by the University of Washington Institutional Animal Care and Use Committee.

## Results

### Characterization of growth, onset of disease, survival, and sex-specific differences in *Ndufs4*^*−/−*^ mice

**Fig. 1:**
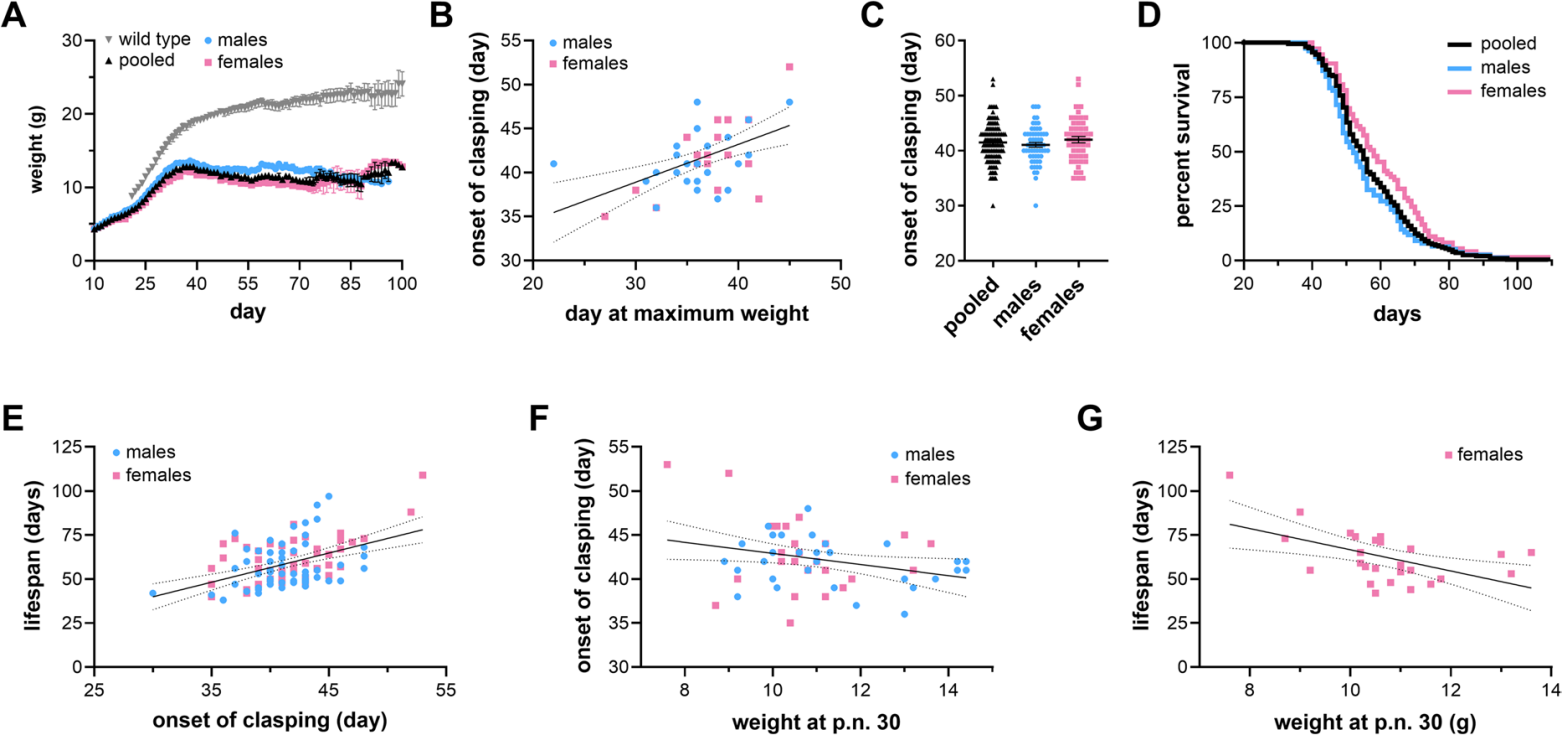
Sex-specific traits of *Ndufs4*^*−/−*^ mice. (**A**) Weight progression in male (blue) and female (pink) *Ndufs4*^*−/−*^ mice and in both sexes pooled (black). Mixed effect model analysis *p*: 0.0015. (**B**) Correlation between onset of clasping and maximum weight in male (blue) and female (pink) *Ndufs4*^*−/−*^ mice. Simple linear regression, *p* < 0.001, both sexes.** (C)** Onset of clasping in male (blue), female (pink) *Ndufs4*^*−/−*^ mice and in both sexes pooled (black) (male = 41.1 ± 3.3 days, female = 42.0 ± 4.1 days, *p* = 0.18, one-way ANOVA). (**D**) Kaplan–Meier survival plot of male (blue) and female (pink) *Ndufs4*^*−/−*^ mice and in both sexes pooled (black). Log-Rank *p* = 0.025 males vs. females. (**E**) Correlation between onset of clasping and survival in male (blue) and female (pink) *Ndufs4*^*−/−*^ mice. Simple linear regression *p* < 0.001, both sexes. (**F**) Correlation between weight at post-natal day 30 (p.n. 30) and onset of clasping in male (blue) and female (pink) *Ndufs4*^*−/−*^ mice. Simple linear regression *p* = 0.0329, both sexes. (**G**) Correlation between weight at post-natal day 30 (p.n. 30) and lifespan in female *Ndufs4*^*−/−*^ mice. Simple linear regression *p* = 0.0051

To probe the sex-specific effects of a complex I deficiency on disease outcome, we first performed an unbiased retrospective analysis of the accumulated data from our *Ndufs4*^*−/−*^ mouse colony over the past ten years. *Ndufs4*^*−/−*^ mice are generally smaller than healthy control mice (Fig. 1A). They grow less than wild type littermates with a maximum mean weight of ~ 12.8 g near post-natal (p.n.) day 39. However, *Ndufs4*^*-/-*^ mice display relatively normal neurobehavioral phenotypes early in life and show a sexual dimorphism in body size between male and female animals (Fig. 1A). Weight peaks at 37 ± 9 days (p.n. 38 males, p.n. 36 females) which generally correlates with the onset of neurological symptoms (Figure S1A, Pearson R 0.604, *p* < 0.0001). Weight loss is associated with the appearance of neurological lesions and astrogliosis that increases in severity over time [5, 8].

A well-established and easily observable phenotype that strongly correlates with brain damage in these mice is the onset of a trunk curl (clasping). This is recognized as when the *Ndufs4*^*−/−*^ mouse trunk begins to curl upward while it is suspended by the tail. This is most severe in the early stages of trunk curling, with the front paws often being above the mouse’s hindlimbs. It is unreliable to score the severity of trunk curling, and thus, we determine whether it is simply absent or present. Helicoptering, circling, limb grasping, paw clasping, and rolling are also commonly observed neurologic complications that occur near the onset of clasping. These phenotypes normally arise 5 ± 4 days after maximum weight is reached in *Ndufs4*^*−/−*^ mice (Fig. 1B). Weight generally declines even further from this point until endpoint or natural death. The average onset of clasping in our colony is 41.5 ± 3.7 days when mice are fed standard chow diets (Fig. 1C).

The average lifespan of this short-lived model in our colony is 56.9 ± 12.2 days with a median lifespan of 55 days using a standard chow (Fig. 1D). There is a strong correlation between the onset of clasping and lifespan in both sexes (Fig. 1E, Figure S1A, S1B, S1C). Similarly, weight at p.n. day 30 correlates negatively with the onset of clasping in both sexes (Fig. 1F). In female mice, there is also a strong negative correlation between weight at p.n. day 30 and lifespan, as well as strong positive correlations between the day maximum weight is reached and lifespan or onset of neurological symptoms (Fig. 1G, Figure S1C). No such correlations were identified when comparing lifespan to weight at weaning, litter size, litter number, or cage occupancy.

We next separately evaluated disease progression and survival data for female and male mice. Despite no differences in the onset of clasping (Fig. 1C), females exhibit increased lifespans (Fig. 1D). Notably, females live ~ 5 days longer than males after the onset of neurobehavioral symptoms (i.e., onset of clasping), suggesting females have increased resilience to mitochondrial disease fatality despite no known differences in the onset of disease. This difference represents a considerable ~ 10% extension in lifespan of female *Ndufs4*^*−/−*^ vs. male cohorts. The origin of these sex-specific differences remains unknown, though they are consistent with known sexual dimorphism in longevity and susceptibility to age-related pathologies across multiple species of mammals [16].

### Temperature does not affect survival in* Ndufs4*^*−/−*^mice

**Fig. 2:**
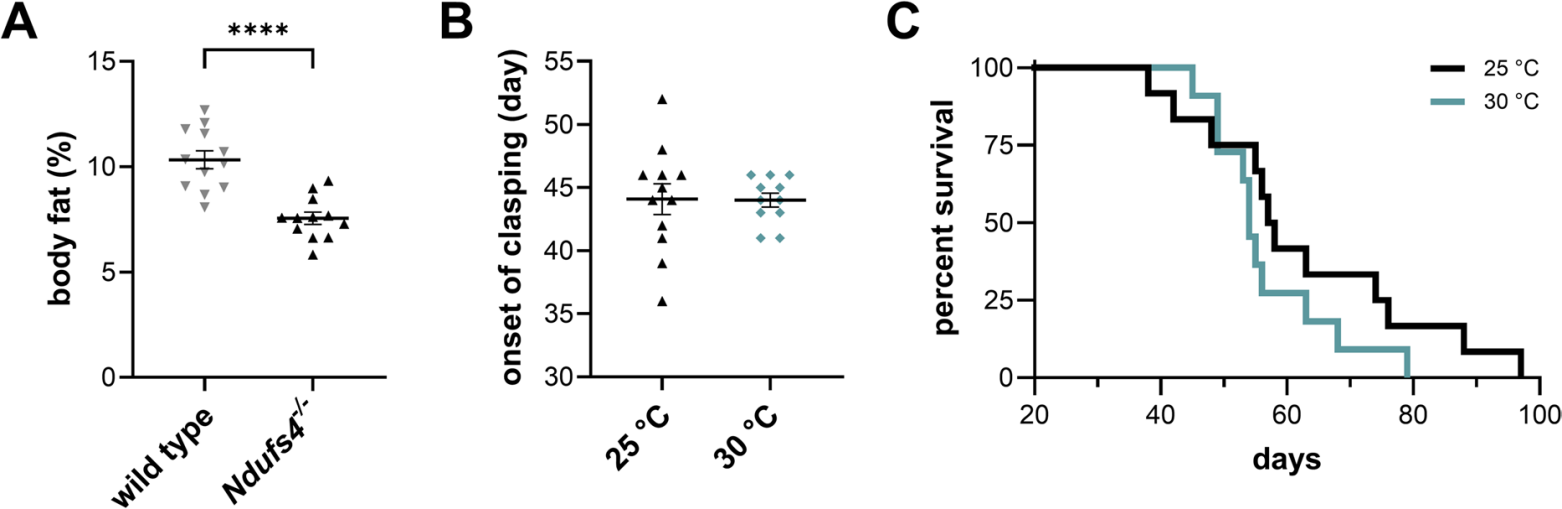
Thermoneutrality does not influence survival in *Ndufs4*^*−/−*^ mice. (**A**) Body composition at p.n. day 50 in pooled wild-type (gray) and *Ndufs4*^*−/−*^ (black) mice. ****p < 0.0001 unpaired *t* test.(**B**) Onset of clasping and (**C**) Kaplan–Meier plot of *Ndufs4*^*−/−*^ mice raised at 25 °C (black) or 30 °C (teal). Log-Rank p > 0.05

There is accumulating evidence that female animals exhibit more robust mitochondrial function compared to males. Reports suggest females have increased mitochondrial biogenesis and improved mitochondrial function as assessed by common readouts such as respiration, oxygen saturation, hydrogen peroxide formation, antioxidant levels, and other factors [17, 18]. Healthy female mice additionally have higher levels of body fat and are generally smaller than age-matched male cohorts. The *Ndufs4*^*−/−*^ mice progressively lose body fat and experience decreased core body temperatures or increased hypothermic events as disease progresses [19]. These mice appear thin with ~ 50% reduced body fat near median lifespan compared to wild-type (WT) controls (~ 7.5% body fat for *Ndufs4*^*−/−*^ mice and > 10.3% for WT mice) (Fig. 2A).

Inhibition of mTOR with rapamycin increases survival and similarly increases abundance of thermoregulatory genes in *Ndufs4*^*−/−*^ mice [20]. We thus asked whether *Ndufs4*^*−/−*^ mice thrive when housed in a thermoneutral environment where there is no additional energy expenditure required to maintain core body temperature. We housed *Ndufs4*^*−/−*^ male and female mice in an environmental chamber kept at 30 °C and compared to mice housed at room temperature. We did not observe any differences in the onset of clasping (44.0 ± 1.8 days at 30 °C, Fig. 2B) and no differences in the lifespan (*p* = 0.2065, log-rank Fig. 2C) between *Ndufs4*^*−/−*^ mice housed at 30 °C vs. room temperature, suggesting that the decreases in core body temperature of *Ndufs4*^*−/−*^ mice are secondary to disease pathogenesis.

### 17-alpha-estradiol increases lifespan and delays symptoms of disease in* Ndufs4*^*−/−*^mice

Previously, we showed that disease progression and survival in *Ndufs4*^*−/−*^ mice can be profoundly delayed by rapamycin [7] and acarbose [8], the two most potent drugs identified to extend lifespan in genetically heterogeneous HET3 mice by the National Institute on Aging Intervention Testing Program (ITP) [7, 8]. This evidence led us to investigate whether other positive hits in the list of treatments tested by the ITP are effective at mitigating symptoms of disease in this mouse model.

**Fig. 3:**
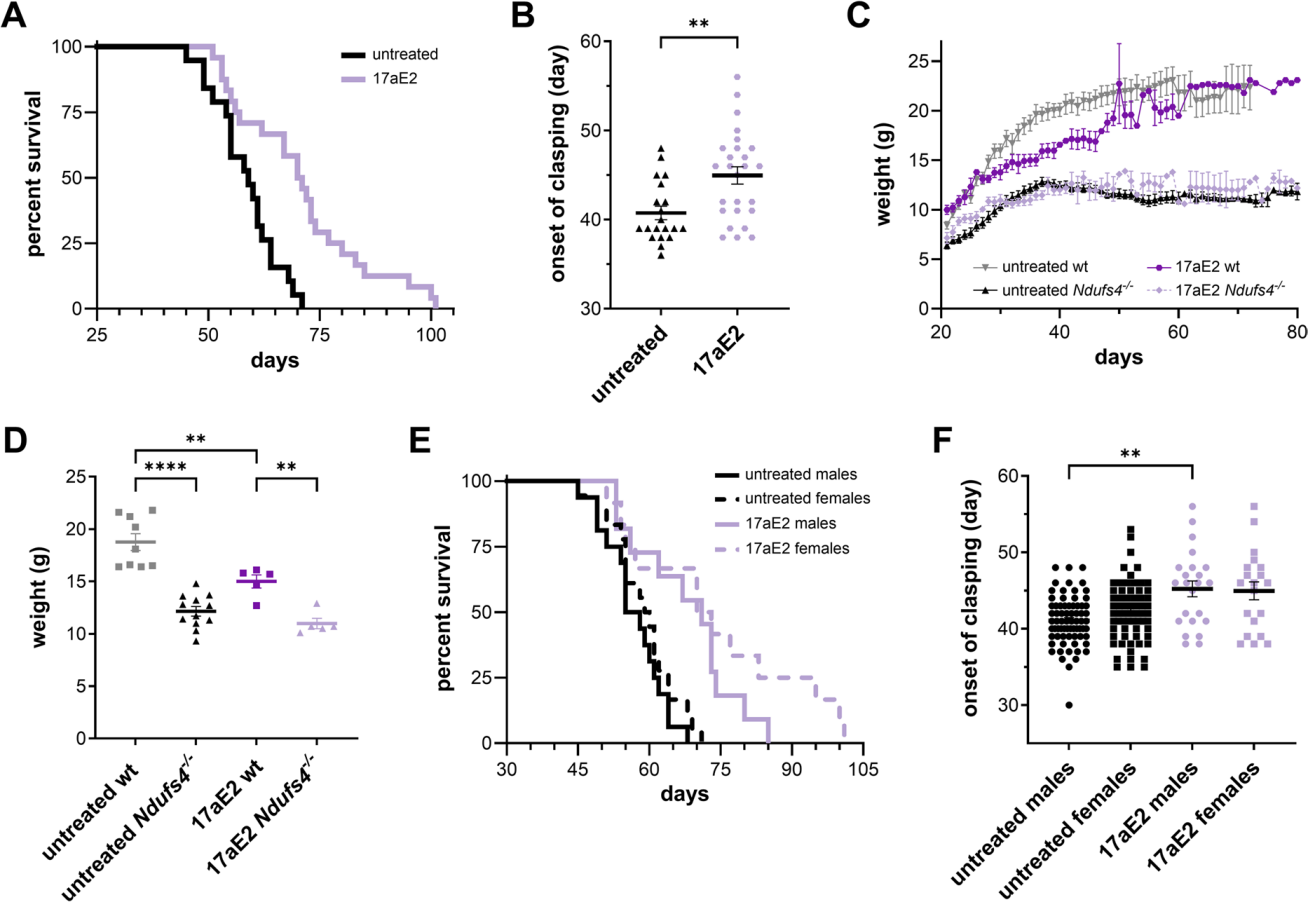
17-alpha-Estradiol (17aE2) increases survival and delays the onset of disease symptoms in *Ndufs4*^*−/−*^ mice. (**A**) Kaplan–Meier plot of untreated (black) or 17aE2-treated (purple) *Ndufs4*^*−/−*^ mice. Log-Rank *p* = 0.0002. (**B**) Onset of clasping in untreated (black) and 17aE2 (purple)-treated *Ndufs4*^*−/−*^ mice. (**C**) Weight progression and (**D**) weight at p.n. day 35 of untreated wild-type (gray), untreated *Ndufs4*^*−/−*^ (black), 17aE2-treated wild-type (dark purple), and 17aE2-treated *Ndufs4*^*−/−*^ (light purple) mice. **p < 0.01, ****p < 0.0001, one-way ANOVA. (**E**) Kaplan–Meier plot of untreated (black) and 17aE2-untreated (purple) male (full line) and female (dotted line) *Ndufs4*^*−/−*^ mice. Log-Rank *p* = 0.0019 untreated males vs. 17aE2 males; Log-Rank *p* = 0.0020 untreated females vs. 17aE2 females. (**F**) Onset of clasping in untreated (black) or 17aE2-treated (purple) male (circle) and female (squares) *Ndufs4*^*−/−*^ mice. **p = 0.002 Brown-Forsythe and Welch ANOVA

17-alpha-estradiol (17aE2) extends average lifespan in male HET3 mice by 19% at a dose of 14.4 ppm in their diet [21, 22], making it the third most effective intervention to arise from the ITP. To determine whether 17aE2 extends survival and delays onset of disease in *Nduf4*^*−/−*^ mice, we fed mutant and wild-type littermates chow containing 14.4 ppm 17aE2 from weaning (p.n. day 21) until the end of life. *Ndufs4*^*−/−*^ mice-fed 17aE2 had a 19.5% increase in survival (70.5 vs. 59 days median) and a 10% delay (46 vs. 39 days median) in the onset of neurological symptoms (Fig. 3A, B). We examined the effects of 17aE2 on body weight in *Ndufs4*^*−/−*^ and wild-type littermates. Similar to what is seen in HET3 mice [23], 17aE2 reduced body weight in both wild-type and *Ndufs4*^*−/−*^ mice compared to untreated animals (Fig. 3C, D). We did not detect any substantial difference in survival between male and female mice treated with 17aE2 (71 vs. 71.5 days median, Fig. 3E); however, when compared to same-sex untreated animals in our historical cohort, only male *Ndufs4*^*−/−*^ treated with 17aE2 showed a significant delay in the onset of neurological symptoms (Fig. 3F).

## Discussion

In this paper, we describe prominent features of the *Ndufs4*^*−/−*^ mouse model that make it amenable for aging studies. We identified previously overlooked sexual dimorphism in body size and survival (Fig. 1) along with a wide variability in disease-free survival, similar to the variability observed with disease and frailty in wild-type animals [16]. Notably, this observation is limited to the specific housing and husbandry conditions in one animal vivarium, as well as one background strain, and may not be recapitulated in other studies, similar to the high variability observed in the parental C57Bl6 background [24].

Sex-specific differences also underlie many age-related disorders, as evidenced by both epidemiological studies in patients with AD and in many related mouse models [25–28]. For example, there is literature precedent that dementia in male AD patients progresses more severely and men with AD succumb to disease at faster rates than female patients [28]. This relationship may contribute to the higher prevalence of AD in female populations. In support of this, incidence frequency between men and women is relatively unchanged [29]. This suggests that while male and female AD patients have similar onsets of disease, women are more resilient. Mitochondrial dysfunction is increasingly believed to be critical in AD etiology. However, it is unclear if mitochondrial dysfunction is the root cause of our observed sexual dimorphisms. There may be shared mechanisms of resilience as observed in this model, in mouse models of AD or related dementias, or in the human population. It thus suggests the *Ndufs4*^*−/−*^ mouse model may also be useful to provide insights into age-related neurodegenerative diseases or in the discovery of effective interventions for AD.

We describe a positive correlation between the onset of disease and survival in both sexes (Fig. 1). Although these symptoms are neurological in nature and manifest as neuromotor impairments, their onset and severity can be manipulated by longevity interventions, such as acarbose [8], rapamycin [7, 30], alpha-ketoglutarate [9], oxygen tension [10, 11], and 17aE2 (this study, Fig. 3). These treatments have whole-body encompassing effects on animal physiology and pathology in normative aging. Thus, we propose that the onset of neurological phenotypes in *Ndufs4*^*−/−*^ mice could be used as a proxy for loss of healthspan and the entrance in a “geriatric” state of decline for these animals. One notable exception is the NAD^+^ precursor nicotinamide mononucleotide (NMN), which appears to increase longevity in this model without delaying the onset of neurological symptoms [9]. Notably, we have been unable to see significant effects with either NMN or nicotinamide riboside (NR) on survival and onset of disease in these animals (data not shown). This discrepancy may be due to multiple factors, including dosage, route, and time of administration, as well as the source and storage of these drugs, and further studies are necessary to tease out this difference. Remarkably, NR failed to extend lifespan in HET3 mice [31], suggesting that the longevity effects of NAD precursors may be limited to specific cases and scenarios [32–35]. NMN is unable to cross the blood–brain barrier and act directly in the brain. However, increased longevity in *Ndufs4*^*−/−*^ mice treated with NMN suggests that multiple, systemic factors influence survival in this model, similar to normative aging.

In support of this hypothesis, acarbose appears to rescue disease symptoms and survival by acting on the intestinal microbiome rather than on the central nervous system directly [8], similar to its effects in normative mouse aging [36]. Furthermore, both rapamycin and acarbose have profound effects on liver metabolism that may contribute to delayed onset and progression of disease [7, 37] (unpublished). Lastly, liver-specific ablation of ribosomal protein S6 kinase 1 (S6 K1) also improves disease phenotypes and survival in this model, while brain-specific knockout has no appreciable effects, further supporting the hypothesis that longevity interventions have systemic effects in this model [38].

*Ndufs4*^*−/−*^ mice begin showing signs of disease around p.n. day 35–40. Prior to the onset of disease, weight at p.n. day 30 correlates negatively with overall survival in female *Ndufs4*^*−/−*^ mice (Fig. 1). This is reminiscent of data from non-transgenic mouse models and other species such as dogs, where smaller individuals tend to be longer lived than larger ones [39, 40]. It is unclear as to why this correlation is maintained only in female animals in the *Ndufs4*^*−/−*^ model; in male mice, the effects of body size may be masked by the increased susceptibility to the disease. Further studies are needed to tease out these sex-specific differences.

We and others presumed that thermoregulation is a significant issue in the management of *Ndufs4*^*−/−*^ mice due to their low body fat and depressed body temperature. Some studies suggest that the improper expression of cold shock–associated proteins may be involved in the pathophysiology of disease in *Ndufs4*^*−/−*^ mice [20, 41]. Further, the heightened basal metabolism of mice compared to humans suggests that their optimal housing temperature is 30 °C [42, 43]. Other studies strongly refute the importance of thermoneutrality [44], and thus, it remains unclear. Our data show that *Ndufs4*^*−/−*^ mice experience no benefits when housed at 30 °C when initiated at ages before resting their body temperature drops (Fig. 2B and C). This suggests that impairments in thermoregulation and adipose tissue physiology in *Ndufs4*^*−/−*^ mice are secondary to the pathogenic drivers of disease outcome and the sex-specific effects on lifespan that we observed.

17aE2 extends survival and delays onset of disease in the *Ndufs4*^*−/−*^ mice (Fig. 3). Notably, 17aE2 appears to have a similar magnitude of effect in *Ndufs4*^*−/−*^ mice and HET3 mice and delays the onset of neurological symptoms specifically in male mice, consistent with its sex-specific effects on longevity in wild-type animals. However, we did not identify any major sex-specific effects on survival in *Ndufs4*^*−/−*^ mice. This is consistent with previous observations showing no sex specificity to rapamycin, acarbose, or hypoxia [7, 8, 11], despite clear sexual dimorphism in body size and longevity in this model. Notably, the NIA ITP begins treatment at 6 months of age, when mice are fully developed and in adulthood, whereas *Ndufs4*^*−/−*^ mice never reach sexual maturity due to the severity of the disease. Thus, any sex-specific effect related to sexual hormones and reproductive function may be missed in this model.

To date, three compounds identified by the NIA ITP show longevity benefits in the *Ndufs4*^*−/−*^ mice: rapamycin, acarbose, and 17-alpha-estradiol. Considering that these drugs have very different targets and mechanisms of action, it is surprising that all three of them are effective in the same disease model. We hypothesize that a shared characteristic of these interventions is their ability to influence one or more of the fundamental biological processes behind organismal aging [2, 45, 46], such as mitochondrial dysfunction. Though direct evidence is not available for acarbose and 17aE2, none of these compounds appear to restore oxidative metabolism to the level of wild-type animals in *Ndufs4*^*−/−*^ mice [7]. However, the effects of mitochondrial dysfunction are clearly mitigated by longevity interventions in this model, with positive repercussions on other fundamental hallmarks of aging such as inflammation, nutrient sensing, and dysbiosis. Further studies will determine whether other aging hallmarks may be altered in this mouse model and rescued by longevity interventions. As we previously suggested, aging may be understood as a chronic, acquired mitochondrial disease [47, 48]. While this hypothesis requires further testing, it is evident from this and previous studies that the *Ndufs4*^*−/−*^ mouse may be construed as a model to provide insights for aging-relevant mitochondrial dysfunction, if not normative aging altogether.

In conclusion, we present a very short-lived transgenic model of mitochondrial disease that appears incredibly consistent at recapitulating the effects of longevity interventions in wild-type mice. We propose that the *Ndufs4*^*−/−*^ mouse represents a relatively convenient and inexpensive option to test longevity interventions and dissect their mechanism of action in a complex mammal with lifespan comparable to widely used invertebrate models such as *C. elegans* and *D. melanogaster*. We hypothesize that insights gathered in this model may translate to a normative aging context, generate new hypotheses, and identify new interventions that may have significant impact in both the clinical treatment of mitochondrial disorders and human longevity.

## Supplementary Information

Below is the link to the electronic supplementary material.

Supplementary file1 (JPG 208 KB)

## Data Availability

Any relevant data in addition to that presented in this submission will be made available on public sources (e.g., Dryad) or provided upon reasonable request.
