# Supplementary figures and images for: Ndufs4−/− mice: a testing ground for longevity interventions
Source: GeroScience. 2025 Jun 5;47(5):6129–38. doi: 10.1007/s11357-025-01704-8 (PMC12634981; doi:10.1007/s11357-025-01704-8)

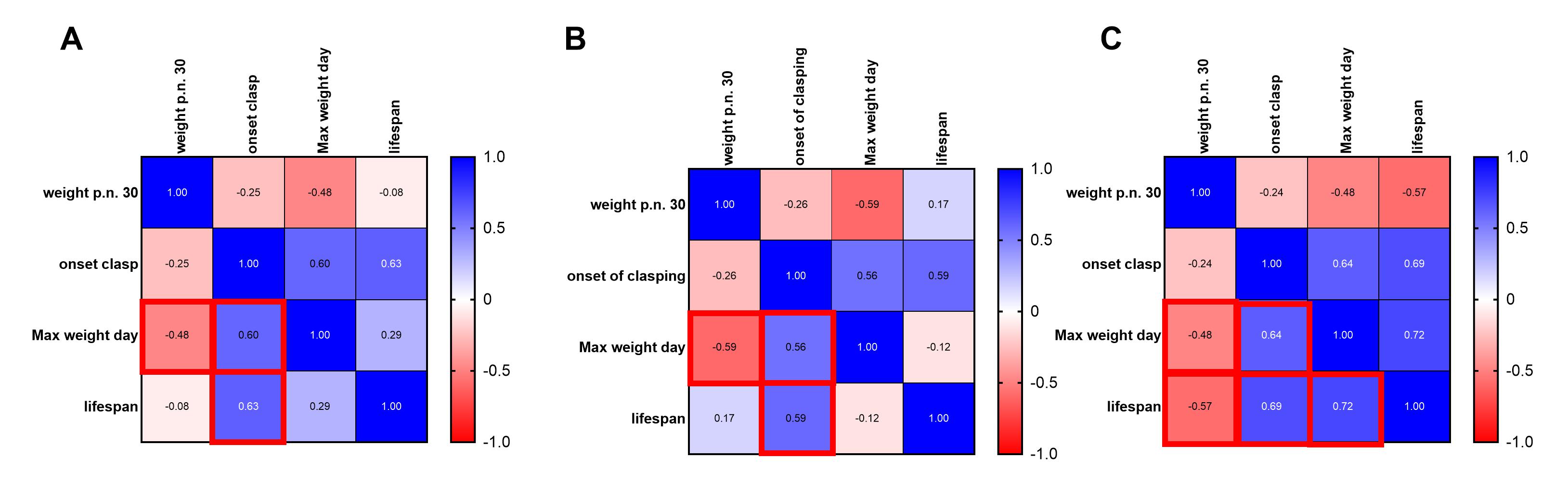

Supplement: Supplementary file 1 — Supplementary file1 (JPG 208 KB) [file 11357_2025_1704_MOESM1_ESM.jpg]
